# Questions on ‘Intervention effects of a kindergarten-based health promotion programme on obesity related behavioural outcomes and BMI percentiles’

**DOI:** 10.1016/j.pmedr.2019.101022

**Published:** 2019-11-21

**Authors:** Colby J. Vorland, Andrew W. Brown, Chanaka N. Kahathuduwa, John A. Dawson, Nana Gletsu-Miller, Theodore K. Kyle, Lehana Thabane, David B. Allison

**Affiliations:** Department of Applied Health Science, Indiana University School of Public Health-Bloomington, Bloomington, IN, USA; Department of Applied Health Science, Indiana University School of Public Health-Bloomington, Bloomington, IN, USA; Department of Psychiatry, School of Medicine, Texas Tech University Health Sciences Center, Lubbock, TX, USA; Department of Nutritional Sciences, Texas Tech University, Lubbock, TX, USA; Department of Applied Health Science, Indiana University School of Public Health-Bloomington, Bloomington, IN, USA; ConscienHealth, Pittsburgh, PA, USA; Department of Health Research Methods, Evidence, and Impact, McMaster University, Hamilton, Ontario, Canada; Department of Epidemiology and Biostatistics, Indiana University School of Public Health-Bloomington, Bloomington, IN, USA

Kobel et al. (2019) report results of a cluster randomized trial examining the effectiveness of the “Join the Healthy Boat” kindergarten intervention on BMI percentile, physical activity, and several exploratory outcomes. The authors pre-registered their study (Steinacker et al., 2016) and described the outcomes and analysis plan in detail previously (Kobel et al., 2017), which are to be commended. However, we noted four issues that some of us recently outlined in a paper on childhood obesity interventions: 1) ignoring clustering in studies that randomize groups of children, 2) changing the outcomes, 3) emphasizing results that were statistically significant from a host of analyses, and 4) using self-reported outcomes that are part of the intervention (Brown et al., 2019).

First and most critically, the statistical analyses reported in the article were inadequate and deviated from the analysis plan in the study’s methods article – an error the authors are aware of and had acknowledged (Kobel et al., 2015) after some of us identified it in one of their prior publications about this same program (Li et al., 2015). The authors pre-specified using generalized estimating equation (GEE) models to account for clustering in the study design (Kobel et al., 2017), on which they based their power calculation (Kobel et al., 2017). However, linear regression models are reported in the final analysis (Kobel et al., 2019). As stated by Gomes et al. (2012); “*For the analysis of clinical outcomes, it is recognized that ignoring clustering underestimates statistical uncertainty, encourages incorrect inferences, and can also lead to bias*”; thus, data must be reanalyzed prior to drawing any conclusions. In addition, the authors reported a plan to evaluate their data using intention-to-treat (Kobel et al., 2017), but this was not followed in the analysis (Kobel et al., 2019). A substantial loss of 43% of participants was reported, along with numerous missing values for each outcome, and these losses likely differentially affect participant characteristics post-randomization and may significantly bias estimates when not appropriately accounted for (George et al., 2016).

Second, the authors switched their primary and secondary outcomes from their original plan. In both the pre-registration and published protocol, the primary outcomes as listed include physical activity/energy expenditure, and secondary outcomes include BMI (Steinacker et al., 2016, Kobel et al., 2017), yet these are reversed in the final article (Kobel et al., 2019). BMI as a percentile is reported in the final article but is not explicitly pre-registered, while anthropometric measurements are listed as secondary outcomes in the registration. An additional outcome of endurance capacity is also reported but not pre-registered.

Third, while the authors focus on an effect of the intervention of p ≤ 0.04 in the abstract, controlling for migration background in their full model raised this to p = 0.153. Because inclusion or exclusion of migration background does not appear to be a pre-specified analytical decision, this selective reporting in the abstract amounts to spinning of the results to favor the intervention (Boutron and Ravaud, 2018).

Fourth, “physical activity and other health behaviours … were assessed using a parental questionnaire.” Given that these variables were also part of the intervention itself, with the control having “no contact during that year,” subjective evaluation may have resulted in differential, social-desirability bias, which may be of particular concern in family research (Havermans et al., 2015). Although the authors mention this in the limitations, the body of literature demonstrating the likelihood of these biases invalidating the measurements raises the question of whether they should be used at all.

Pre-specification of study design, outcomes, and analytic decisions are good scientific practice. Deviations from such plans should be faithfully reported for transparency and interpretation. We request that Kobel et al. reanalyze their data to account for clustering in their design and update their published methods and results to account for deviations from pre-specifications, and to make clear other factors that may potentially undermine the validity of their results.

## Funding

Supported in part by the Gordon and Betty Moore Foundation and NIH grant R25HL124208. The opinions expressed are those of the authors and do not necessarily represent those of the NIH or any other organization.

## Disclosures

Dr. Allison has received personal payments or promises for same from: American Society for Nutrition; American Statistical Association; Biofortis; Columbia University; Fish & Richardson, P.C.; Frontiers Publishing; Henry Stewart Talks; IKEA; Indiana University; Laura and John Arnold Foundation; Johns Hopkins University; Law Offices of Ronald Marron; MD Anderson Cancer Center; Medical College of Wisconsin; National Institutes of Health (NIH); Sage Publishing; The Obesity Society; Tomasik, Kotin & Kasserman LLC; University of Alabama at Birmingham; University of Miami; Nestle; WW (formerly Weight Watchers International, LLC). Donations to a foundation have been made on his behalf by the Northarvest Bean Growers Association. Dr. Allison is an unpaid member of the International Life Sciences Institute North America Board of Trustees. Dr. Allison’s institution, Indiana University, has received funds to support his research or educational activities from: NIH; Alliance for Potato Research and Education; American Federation for Aging Research; Dairy Management Inc; Herbalife; Laura and John Arnold Foundation; Oxford University Press. Dr. Allison's prior institution, the University of Alabama at Birmingham, received gifts, contracts, and grants from the Coca-Cola Company, Pepsi, and Dr. Pepper/Snapple. In the last 12 months, Dr. Brown has received travel expenses from the University of Louisville and grants through his institution from Dairy Management, Inc. and the National Cattlemen’s Beef Association. He has been involved in research for which his institution or colleagues have received grants from the Gordon and Betty Moore Foundation, NIH/NHLBI, NIH/NIA, NIH/NIDDK, and Sloan Foundation. Dr. Dawson discloses his employment by Texas Tech University, grants from the Egg Nutrition Center/American Egg Board, and travel expenses from the American Society for Nutrition. Mr. Kyle discloses consultancy with Nutrisystem and Novo Nordisk. Other authors report no disclosures.
